# Seborrheic-like dermatitis and liver dysfunction in an infant: signs of Langerhans cell histiocytosis^☆^

**DOI:** 10.1016/j.abd.2020.08.035

**Published:** 2021-11-25

**Authors:** Daniela Antoniali, Helena Barbosa Lugão, Daniel Elias, Cacilda da Silva Souza

**Affiliations:** Dermatology Division, Department of Internal Medicine, Faculdade de Medicina de Ribeirão Preto, Universidade de São Paulo, Ribeirão Preto, SP, Brazil

Dear Editor,

This report describes the case of a one-year and five-months-old female child, who had desquamation and pruritus on the scalp for 6 months, with no response to topical corticosteroids and antifungal agents. The physical examination showed weight and height deficit (percentile 3 for height and 1 for weight); coalescent papules under hematic and meliceric crusts, on erythematous-desquamative skin on the scalp and the temporal and retroauricular regions (Fig. 1); ulcerated erythematous-infiltrated lesions on the left axilla and vulva and soft palate erosions. The liver was hardened, palpable five cm from the right costal margin. There was no adenomegaly. Complementary tests showed anemia (Hb: 9.8 mg/dL), liver function tests alterations (AST: 136 U/L, ALT: 152 U/L, ALP: 1821 U/L, GGT: 907.3 U/L); ultrasonography showed the liver at the upper limit of normality, heterogeneous echotexture and areas of periportal hyperechogenicity; magnetic resonance cholangiography and bone scintigraphy showed no alterations; histopathology of the skin showed a lichenoid infiltrate of Langerhans cells with diffuse immunohistochemical positivity for CD1a and S100 (Fig. 2). Thus, the diagnosis of Langerhans cell histiocytosis (LCH) with seborrheic-like dermatitis lesions and liver involvement was established. Oncological treatment was initiated with prednisone and vinblastine for 12 weeks, with no improvement of skin lesions. One month after the end of the induction phase, with continuous lesions on the scalp, a new histopathology analysis showed persistence of a lichenoid inflammatory infiltrate of Langerhans cells with diffuse positivity for CD1a, whereas the ultrasonography showed hepatic infiltration and splenomegaly. The continuation of chemotherapy with mercaptopurine, prednisone and vinblastine for 12 months was indicated (currently ongoing).

**Figure 1:**
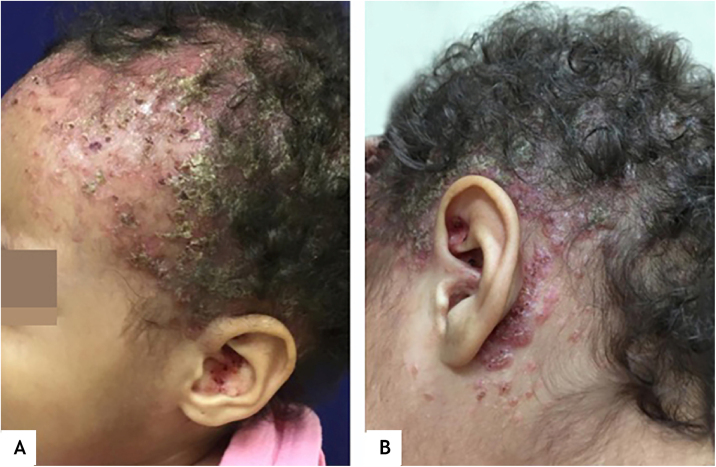
(A), Erythematous-desquamative, infiltrated lesions covered by hematic and meliceric crusts all over the scalp and the ear pinna. (B), Coalescent erythematous papules under hematic and meliceric crusts, extending from the temporal to the retroauricular region.

**Figure 2:**
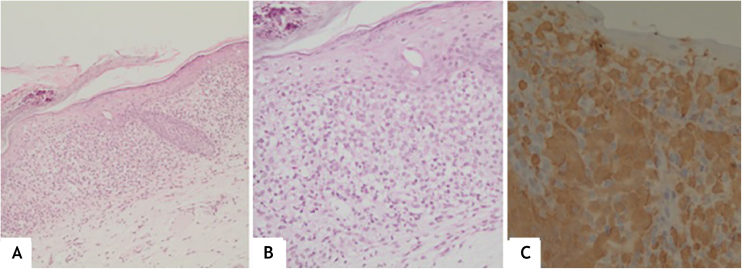
(A), Spongiotic epidermis with foci of basal hydropic degeneration; superficial dermis with moderate diffuse band-like inflammatory infiltrate (Hematoxylin & eosin, ×100). (B), Cells with an oval nucleus, focally lobulated, in addition to mature lymphocytes and some eosinophils (Hematoxylin & eosin, ×200). (C), Diffuse positivity for CD1A (×200).

Historically, LCH has been subdivided into four syndromes (Letterer-Siwe, Hand-Schuller-Christian, eosinophilic granuloma, and Hashimoto-Pritzker); however, current evidence indicates that not all cases fit into these categories.^1^, ^2^ Recently, LCH has been redefined as an inflammatory myeloid neoplasia, attributed to the activation of mutations in the mitogen-activated protein kinase (MAPK) pathway,^2^ with the *BRAF-V600E* gene mutation being the most prevalent one.^3^ Clinically, it is categorized by the involvement of one or multiple systems, either unifocal or multifocal and the presence of involvement of organs at risk (liver, spleen and bone marrow), which define the prognosis and therapeutic response. Single-system LCH has an excellent prognosis, whereas the multi-system form has a mortality risk that requires aggressive treatments.^2^

Although isolated skin disease is rare (2%), cutaneous manifestations are the standard form of presentation in around 80% of cases,^2^, ^3^, ^4^ being the most common in those younger than 2 years-old.^2^ Seborrheic-like dermatitis, erythematous papules, and eczematous lesions are frequent,^4^ among others, including petechiae, purpura, maculae, hypopigmented or umbilicated papules, nodules, vesiculobullous lesions, and pustules,^5^ predominantly on the scalp, abdomen, chest and intertriginous areas. ^4^ The extracutaneous manifestations include lytic bone lesions, diabetes insipidus, growth hormone deficiency, hepatosplenomegaly, and lymphadenopathy.^4^ Liver involvement is seen exclusively in multi-system LCH, presenting as isolated hepatomegaly and/or liver function impairment and jaundice.^5^ The histopathological analysis and positivity for CD1a, S100, and/or CD207 (Langerin) in immunohistochemistry establishes the diagnosis.

Cutaneous manifestations of LCH are variable and may be similar to other prevalent dermatoses. In the presence of intense and refractory seborrheic dermatitis-like condition, LCH should be suspected, and histopathological and multisystem involvement investigations are mandatory.

## Financial support

None declared.

## Authors’ contributions

Daniela Antoniali: Design and planning of the studied case; intellectual participation in the propaedeutic and/or therapeutic conduct of the studied case; review of the literature; drafting and editing of the manuscript.

Helena Barbosa Lugão: Approval of the final version of the manuscript; drafting and editing of the manuscript; participation in research orientation; intellectual participation in the propaedeutic and/or therapeutic conduct of the studied case; critical review of the literature; critical review of the manuscript.

Daniel Elias: Approval of the final version of the manuscript; drafting and editing of the manuscript; participation in research orientation; intellectual participation in the propaedeutic and/or therapeutic conduct of the studied case; critical review of the literature; critical review of the manuscript.

Cacilda da Silva Souza: Approval of the final version of the manuscript; drafting and editing of the manuscript; collection, analysis, and interpretation of data; intellectual participation in the propaedeutic and/or therapeutic conduct of the studied case; critical review of the literature; critical review of the manuscript.

## Conflicts of interest

None declared.
